# Spherical CaCO_3_: Synthesis, Characterization, Surface Modification and Efficacy as a Reinforcing Filler in Natural Rubber Composites

**DOI:** 10.3390/polym15214287

**Published:** 2023-10-31

**Authors:** Khansinee Longkaew, Alain Gibaud, Wasan Tessanan, Philippe Daniel, Pranee Phinyocheep

**Affiliations:** 1 Department of Chemistry, Faculty of Science, Mahidol University, Rama VI Road, Payathai, Bangkok 10400, Thailand; khansinee.lon@gmail.com (K.L.); t.wasan18@gmail.com (W.T.); 2 Institute of Molecules and Materials of Le Mans (IMMM), UMR CNRS 6283, Avenue Olivier Messiaen, CEDEX 9, 72085 Le Mans, France; alain.gibaud@univ-lemans.fr (A.G.); philippe.daniel@univ-lemans.fr (P.D.)

**Keywords:** calcium carbonate, spherical shape, natural rubber, reinforcing filler, surface treatment

## Abstract

Natural rubber (NR), an important natural polymer derived from the *Hevea brasiliensis* tree, has been widely used in the rubber industry owing to its excellent elastic properties. However, it requires reinforcing fillers to improve its mechanical properties for the manufacturing of rubber products. Generally, calcium carbonate (CaCO_3_) is employed as a non-reinforcing filler. This work aimed to synthesize spherical-shaped CaCO_3_ at a submicrometric scale without and with surface treatment and explore its utilization as a reinforcing filler in NR composites. The morphological shape and polymorphic phase of CaCO_3_ were investigated using SEM, TEM, XRD, ATR-FTIR and Raman techniques. The mechanical properties of various amounts (0 to 60 phr) of CaCO_3_-filled NR composites were explored. As a result, the NR/treated CaCO_3_ composites provided higher tensile strength than the NR/untreated CaCO_3_ composites and pure NR at all filler loadings. This may have been due to the improved interfacial interaction between NR and CaCO_3_ with the improved hydrophobicity of CaCO_3_ after treatment with olive soap. The optimal filler loading was 20 phr for the highest tensile strength of the rubber composites. In addition, the elongation at break of the NR/treated CaCO_3_ was slightly decreased. Evidence from SEM and FTIR revealed the vaterite polymorph and shape stability of CaCO_3_ particles in the NR matrix. The results demonstrate that the particle size and surface treatment of the filler have essential effects on the mechanical property enhancement of the rubber composites. Synthesized spherical CaCO_3_ could be a potential reinforcing filler with broader application in polymer composites.

## 1. Introduction

Natural rubber (NR) is a valuable natural elastomer that is obtained in colloidal form from the rubber tree *Hevea brasiliensis*. Rubber particles are dispersed in water in the latex form of 30% dry rubber content (DRC). In general, NR latex is concentrated into 60% DRC for commercial applications. The chemical structure of NR is that of a non-polar hydrocarbon with a cis-1,4-polyisoprenic structure [1,2,3]. It possesses excellent elastic properties and good mechanical properties. However, NR latex or solid NR must be mixed with curing chemicals and reinforced by fillers to improve its mechanical properties, including tensile strength, elongation, modulus, and tear resistance [4,5]. Normally, fillers are divided into reinforcing, semi-reinforcing, and non-reinforcing categories [5,6]. The reinforcing efficiency of a filler in a polymer depends on the particle size, shape, specific surface area, and compatibility between the polymer matrix and filler [7]. There are several fillers used in the rubber industry depending on the target application, such as carbon black [8,9,10], precipitated silica (SiO_2_) [11], calcium carbonate (CaCO_3_) [12,13], clay [14], talc [15], etc. Carbon black is one of the most widely used reinforcing fillers in the automotive industry, especially tire manufacturing, and has the potential to improve the strength of rubber due to its nanoscale particle size (<100 nm) [5]. Silica is incorporated into rubber, particularly for tire treads, to lower the rolling resistance. However, owing to the polarity difference between silica and rubber, modification of the silica surface is required to reduce its polarity and enhance the composite performance. In contrast, CaCO_3_ is mostly used as a semi-reinforcing or non-reinforcing filler [5]. This is due to the major drawback of CaCO_3_ having a particle size larger than 100 nm. It is therefore useful as a cost-effective dilution filler and for processability for some applications [16]. Consequently, many attempts have been made to reduce the particle size of CaCO_3_ to the submicrometric or nanometric scale to enhance its degree of reinforcement [17,18,19,20].

CaCO_3_ is an abundant inorganic material that can be obtained from nature [21,22] or by chemical synthesis [23,24,25]. Among many chemical syntheses, the simplest method of CaCO_3_ synthesis is solution precipitation from a mixture of calcium ions (Ca^2+^) and carbonate ions (CO_3_^2−^). The crystal shape and polymorph of the CaCO_3_ particle can be changed by adjusting various parameters, such as the concentration of reactants, mixing temperature, mixing speed, and organic or aqueous medium. The crystal polymorphs of CaCO_3_ are known as vaterite, aragonite, and calcite. The different polymorphic phases exhibit different shapes. Vaterite has a typical hexagonal shape, demonstrating a spherulitic or framboid morphology [26], while aragonite demonstrates an orthorhombic or needle-like shape [27,28]. The most thermodynamically stable phase is calcite, which has a typical trigonal morphology, showing a cubical shape [29,30]. An aqueous medium containing hydroxyl groups, such as glycerol, ethylene glycol, or sucrose, is the key factor in increasing the viscosity of the mixing system, influencing the decrement in particle size [17,20]. Using sucrose as the aqueous medium in the mixing procedure can reduce the particle size of CaCO_3_ to the submicron scale, with a spherical shape and homogeneous distribution [20]. Moreover, CaCO_3_ can be incorporated in the NR latex stage and maintain its vaterite polymorph. Q. Fang and coworkers investigated different shapes, namely spherical, chain-shaped, and cubic, of CaCO_3_ in NR vulcanizates [12]. The results showed that spherical-shaped CaCO_3_, having a high specific surface area, exhibited the best mechanical properties for loaded NR composites compared with chain-shaped and cube-shaped CaCO_3_. However, the authors did not show the properties of unfilled NR vulcanizates. The particle shape and size of the filler are not the only key factors in the reinforcing efficiency: the interaction between the matrix and filler of the polymer composite also plays an important role [12,31,32]. To improve the reinforcing efficacy of CaCO_3_ in an NR composite, the interfacial interaction between the two phases can be enhanced by adding a compatibilizer to the CaCO_3_-filled NR or modifying the surface of hydrophilic CaCO_3_ to provide it with a hydrophobic character. It was reported that the addition of lauryl alcohol to NR incorporated with CaCO_3_ resulted in improved CaCO_3_ dispersion in the NR matrix, hence increasing the tensile strength of the rubber composite [33]. The surface modification of hydrophilic CaCO_3_ to produce hydrophobic properties has been explored by several researchers using stearic acid and soaps [34,35,36]. However, the utilization of a hydrophobic CaCO_3_-filled NR composite is rarely found. Accordingly, it is challenging to synthesize submicrometric CaCO_3_ particles with a spherical shape (vaterite polymorph) together with hydrophobic surface modification for further use as a reinforcing agent in a rubber composite.

This work, therefore, was focused firstly on CaCO_3_ preparation using a precipitation process to produce submicron spherical-shaped CaCO_3_ with a high percentage of the vaterite phase. The surface modification of the synthesized vaterite CaCO_3_ using olive soap was also carried out. The morphological shape, particle size, and polymorphic phase of the obtained CaCO_3_ particles were examined by scanning electron microscopy (SEM), transmission electron microscopy (TEM), X-ray diffraction (XRD), Brunauer–Emmett–Teller (BET) analysis, attenuated total reflectance–Fourier transform infrared (ATR-FTIR) spectroscopy, and Raman spectroscopy. The wettability of the particle surface was measured by its optical contact angle. Then, the untreated and treated spherical CaCO_3_ particles were incorporated into NR with varying filler content and compared with the neat NR. The mechanical and thermal properties of NR/untreated CaCO_3_ and NR/treated CaCO_3_ composites were investigated, including phase morphology by SEM.

## 2. Materials and Methods

### 2.1. Materials

The reactants for calcium carbonate (CaCO_3_) synthesis, namely ammonium carbonate ((NH_4_)_2_CO_3_) and calcium chloride dihydrate (CaCl_2_∙2H_2_O), were purchased from Acros Organics (Roissy Charles de Gaulle, Paris, France). Commercial-grade granulated sugar was purchased from Pulverent Sugar Poedersuiker (Picardie, Paris, France). Olive oil was obtained from Carrefour BIO Huile d’Olive Vierge Extra Extraite a Froid (Massy Cedex, France). Reagent-grade sodium hydroxide (NaOH) was obtained from Honeywell (Offenbach, Germany). High-ammonia NR latex with 60% DRC was purchased from the Thai Rubber Latex Corporation (Chonburi, Thailand). Commercial-grade curing agents, specifically ZnO, stearic acid, N-cyclohexyl benzothiazole-2-sulfenamide (CBS), and sulfur, were supplied by Kitpiboon Chemical Ltd. (Bangkok, Thailand).

### 2.2. Methods

#### 2.2.1. Untreated CaCO_3_ Synthesis

The untreated CaCO_3_ powder was prepared following the synthesis procedure from our previous work [20]. Briefly, 50% by weight of sucrose solution was used as an aqueous medium to dissolve 1.0 M of (NH_4_)_2_CO_3_ solution and 1.0 M of CaCl_2_ solution. Both reactants were mixed at high speed (>10,000 rpm) using a blender under 25 ± 1 °C for 60 s. The obtained CaCO_3_ powders were collected by centrifugation at 4200 rpm for 6 min, using an Allegra X-22 Centrifuge (Beckman Coulter, IN, USA). Then, the powder was washed several times with ethanol before drying at 70 °C for 3 h in an oven.

#### 2.2.2. Olive Soap Preparation

The olive soap used for the surface modification of CaCO_3_ was prepared by saponification between olive oil and NaOH. First, 1.3 g NaOH was dissolved in 3.9 mL of DI water, which was then mixed with olive oil (10 g) while stirring at 500 rpm for 3 h at room temperature. The obtained solid soap was dried at room temperature for a few days before further use.

#### 2.2.3. Treated CaCO_3_ Synthesis

Next, 0.4 g of solid olive soap was dissolved in 10 mL of ethanol at 80 °C. Then, the olive soap solution was cooled down to room temperature before adding 100 mL of 1.0 M (NH_4_)_2_CO_3_ dissolved in sucrose solution. The mixture was then added to 100 mL of 1.0 M CaCl_2_ dissolved in sucrose solution, followed by blending at high speed (>10,000 rpm) under 25 ± 1 °C for 60 s. The treated CaCO_3_ was obtained by centrifugation, in the same manner as untreated CaCO_3_.

#### 2.2.4. Preparation of NR/CaCO_3_ Composites

- NR/CaCO_3_ compound preparation

For both the untreated and treated samples, 10, 20, 40, or 60 parts per hundred rubbers (phr) were added to 60% DRC NR latex and mixed by mechanical stirring at 600 rpm for 10 min. The NR/CaCO_3_ latex compounds were poured into aluminum plates and dried at room temperature under an ambient atmosphere for approximately 2 weeks.

- NR/CaCO_3_ composite preparation

The NR/CaCO_3_ composites were prepared following the mixing formulation in Table 1. All ingredients were mixed using a 2-roll mill mixing machine (W100T, Collin GmbH, Mühldorf, Germany) at room temperature. The mixing process was performed as follows: mastication of the NR or NR/CaCO_3_ compounds was performed on the roll for 5 min, and then ZnO and stearic acid were added and mixed for 5 min. Thereafter, the curative agents CBS and sulfur were added and mixed for 5 min. The overall mixing time was kept constant at 15 min. After mixing, 10 end-roll passes were made before sheeting the compound off the 2-roll mill. The neat NR and NR/CaCO_3_ composites were compressed into a sheet of 1 mm thickness by heat pressing at 140 °C, following their optimal cure times (t_c90_), which were obtained using a moving die rheometer (MDR) (Dynisco MDR-CGM, Franklin, MA, USA) operated at 140 °C. The rubber sheets were cut into dumbbell-shaped specimens and stored at room temperature for at least 8 h before testing.

### 2.3. Characterization of CaCO_3_ Fillers and NR/CaCO_3_ Composites

#### 2.3.1. Optical Contact Angle

The contact angle measurement of CaCO_3_ powders was performed using an optical contact angle instrument (SL200KS, Kino, Boston, MA, USA). A water droplet was applied on the surface of the powder following the sessile drop method, with a capture speed of 7 frames per second, and axisymmetric drop shape analysis was performed with the Young–Laplace equation in fitting mode.

#### 2.3.2. Scanning Electron Microscopy (SEM)

The morphology of the samples, namely the CaCO_3_ particle surfaces and tensile-fractured surfaces of NR/CaCO_3_ composites coated with gold, was visualized by SEM (JEOL JSM 65 VL, Peabody, MA, USA) at 20 kV with a 10 mm working distance. The average particle size and distribution of CaCO_3_ particles were determined using the ImageJ software (v1.8.0). In each CaCO_3_ sample, the number of particles examined exceeded 150.

#### 2.3.3. Transmission Electron Microscopy (TEM)

TEM (JEM-2100, Peabody, MA, USA) was performed to further examine the particle surfaces. The obtained CaCO_3_ particles were dispersed in ethanol before being deposited as droplets onto TEM grids coated with a carbon film.

#### 2.3.4. Attenuated Total Reflectance–Fourier Transform Infrared (ATR-FTIR) Spectroscopy

The ATR-FTIR spectra were obtained to study the chemical structure and vibration bands of the filler and rubber composites using Bruker Vertex 70v equipment (Billerica, MA, USA) in the ATR-IR mode, working with 20 scans at a resolution of 1 cm^−1^, in the range of 400–4000 cm^−1^.

#### 2.3.5. Raman Spectroscopy

A Raman microscope (XploRA, Horiba, Lyon, France) was used to characterize the polymorphs of CaCO_3_ particles. The spectra were recorded from 400 to 4000 cm^−1^, using a wavelength of 785 nm and a detector value of 1200 gr/mm.

#### 2.3.6. X-ray Diffraction (XRD) Analysis

XRD analysis of the CaCO_3_ powders, NR, and NR/CaCO_3_ composites was conducted using a Panalytical Empyrean diffractometer (Malvern, UK) with a 2θ range of 20–40°, step size of 0.052°, monochromatic Cu-Kα radiation (λ = 1.54 Å), acceleration voltage of 40 kV, and 30 mA beam current. The crystalline percentage of the CaCO_3_ polymorphs was calculated using the MAUD software (v2.94) based on Rietveld refinement, which was designed especially for calculating both the structural and phase parameters.

#### 2.3.7. Thermogravimetric Analysis (TGA)

Thermogravimetric analysis (TGA) (SDT 296, Universal V3.4C TA instrument, New Castle, DE, USA) was performed to investigate the thermal stability of the CaCO_3_ powders and NR/CaCO_3_ composites, using 10 mg of each sample. The samples were investigated in the heating range of 40–800 °C under a N_2_ atmosphere. A heating rate of 10 °C/min was used for CaCO_3_ powder [37]. As the heating rate can affect the decomposition of a polymer, the heating rate used for polymer composites is generally higher than that for the filler [38]. Thus, the heating rate of 20 °C/min was used for NR/CaCO_3_ composites, referring to NR/cellulose nanocrystal composites [39].

#### 2.3.8. Brunauer–Emmett–Teller (BET) Analysis

The BET-specific surface area in m^2^/g unit was measured using a TriStar II 3020 V1.03 Micromeritics Instrument (Norcross, GA, USA), based on N_2_ adsorption, using a sample mass of 2.5 g.

### 2.4. Mechanical Properties of NR/CaCO_3_ Composites

#### 2.4.1. Tensile Properties

The tensile properties, namely the tensile strength, elongation at break, and 100% (M100) and 300% moduli (M300), of neat NR and NR/CaCO_3_ for all variations of filler loading were determined according to the ASTM D 412-98 [40] standard test using dumbbell-shaped specimens with 1 mm thickness and a type C die for cutting. A tensile machine (Instron 5566, Instron, High Wycombe, UK) was used with a crosshead speed rate of 500 mm/min. The average results were taken from 5 testing specimens.

#### 2.4.2. Hardness (Shore A)

The hardness (Shore A) of rubber vulcanizates with and without filler was evaluated using a hardness tester (Wallace H17A, Surrey, UK) in accordance with the ASTM D2240-97 standard [41]. The specimen thickness was 6 mm. The testing was conducted at different positions on the specimen, and the average hardness value of each specimen was calculated using 6 positions.

#### 2.4.3. Statistical Analysis

An analysis of variance (ANOVA) was performed using the Single Factor program with the Data Analysis tool in Microsoft Excel 2019. The *t*-test method was used for the comparison of two samples, assuming unequal variance to determine the significant differences among the mean values (*p* < 0.05).

## 3. Results and Discussion

### 3.1. Untreated and Treated CaCO_3_ Powders

**Figure 1:**
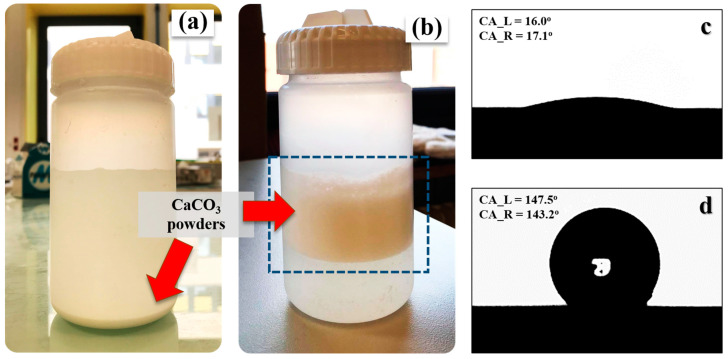
Physical appearance of CaCO_3_ powder in solution before centrifugation: (**a**) untreated CaCO_3_ powder and (**b**) treated CaCO_3_ powder. The contact angle (CA) behavior of (**c**) untreated and (**d**) treated CaCO_3_ powders, presenting CA values for the left (L) and right (R) sides of the droplet.

**Figure 2:**
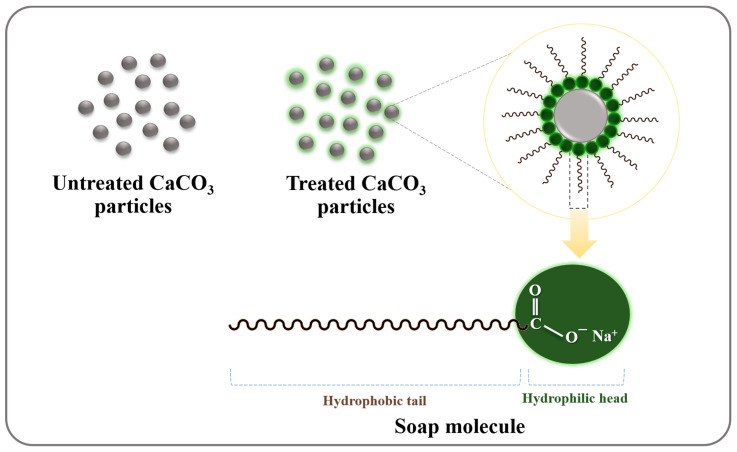
Proposed schematic adsorption of soap molecules on the surface of CaCO_3_ particles.

The features of untreated and treated CaCO_3_ obtained by precipitation in the sugar solution are demonstrated in Figure 1a,b, respectively. Figure 1a shows that, without the olive soap surface treatment, the CaCO_3_ powders settled in the bottom of the container, whereas the treated CaCO_3_ particles floated on the surface of the sugar solution (Figure 1b). This is a simple indication that the surface of the CaCO_3_ was altered. Then, the surface wettability was evaluated by considering the contact angle of the untreated and treated CaCO_3_, as shown in Figure 1c,d, respectively. The average contact angle of untreated CaCO_3_ powder is 16.5°, indicating hydrophilic particles. Conversely, the contact angle of CaCO_3_ powder treated with olive soap is 145.4° (Figure 1d), revealing hydrophobic behavior. The fatty acid composition of olive oil consists of 55–83% oleic acid, 3.5–21% linoleic acid, and 7.5–20% palmitic ester [42]. These fatty acids interact with sodium hydroxide (NaOH) by saponification, producing sodium carboxylate (RCOONa) and glycerol. The formation of soap molecules adsorbed on the surface of CaCO_3_ particles is demonstrated in Figure 2. The soap molecules contain two parts, a hydrophilic head and a hydrophobic tail. The hydrophilic heads are located at the CaCO_3_ surface, while the hydrophobic tails extend out into the surroundings. After the addition of the soap solution into the CaCl_2_ reactant solution, the sodium carboxylate chains could be interchanged with Ca^2+^ ions, forming Ca carboxylate (R(COO^−^)_2_Ca). During the precipitation between Ca^2+^ and CO_3_^2+^ ions, some of the carboxylate chains were adsorbed on the surfaces of the CaCO_3_ particles, resulting in hydrophobic surface behavior. Therefore, the agreement between the physical appearance of the precipitated CaCO_3_ solution and the contact angle confirmed that the surface wettability of CaCO_3_ can be changed from hydrophilic to hydrophobic by precipitation using olive soap.

**Figure 3:**
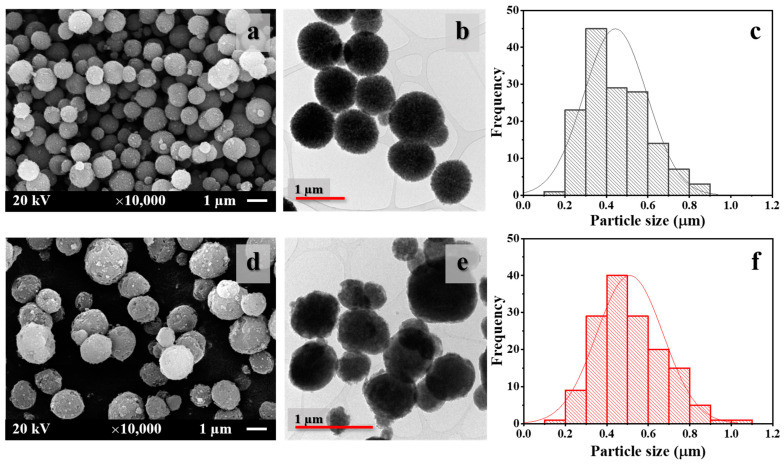
SEM micrographs at 10,000× magnification (1 µm scale bar), TEM micrographs with 1 µm scale bar, and particle size distribution histograms of (**a**–**c**) untreated CaCO_3_ and (**d**–**f**) treated CaCO_3_ powders.

The morphological shape and particle size distribution of the prepared CaCO_3_ particles were investigated by SEM and TEM, as shown in Figure 3. The spherical shape of untreated and treated CaCO_3_ was observed by SEM, as shown in Figure 3a,d, respectively. Both samples also exhibited narrow particle size distributions. The histograms in Figure 3c,e reveal the particle sizes of untreated and treated CaCO_3_ particles, respectively. The particles of untreated CaCO_3_ were on the submicron scale (0.42 ± 0.14 µm), which was due to the use of sucrose as a medium in the precipitation process [18,20]. The addition of olive soap in the precipitation system during CaCO_3_ preparation affected the surface modification of CaCO_3_, as discussed earlier, and increased the particle size of CaCO_3_ (0.52 ± 0.16 µm), as shown in Table 2, although the particles remained in the submicron range.

The TEM micrographs of untreated CaCO_3_ (Figure 3b) and treated CaCO_3_ (Figure 3e) particles were obtained. The surface morphology of CaCO_3_ particles after treatment with olive soap was changed, presenting rougher and larger particles than the untreated particles. This may be attributed to not only the adsorbed soap on the surface of treated CaCO_3_ but also the penetration of soap inside the particles.

The specific surface area of the untreated and treated CaCO_3_ was analyzed by the BET technique. The BET values shown in Table 2 reveal that the specific surface area of untreated CaCO_3_ powders (39.32 m^2^/g) was higher than that of the treated CaCO_3_ powders (34.45 m^2^/g). The lower surface area is consistent with the larger particle size of treated CaCO_3_ particles. Moreover, the obtained BET values indicate that the CaCO_3_ particles were porous, which is typical and unique to the vaterite polymorph.

**Figure 4:**
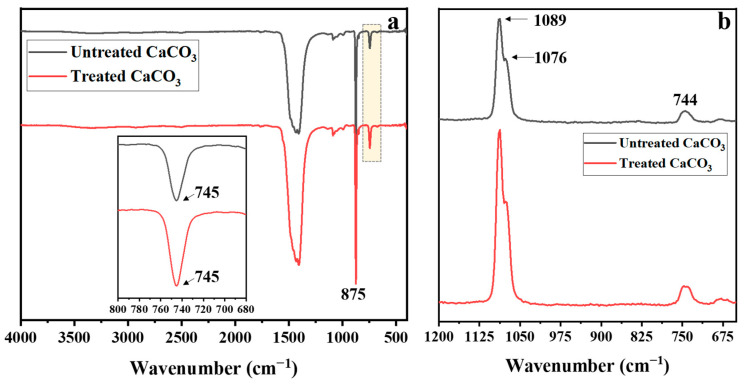
(**a**) ATR-FTIR spectra in the wavenumber range of 400–4000 cm^−1^ and (**b**) Raman spectra (650–1200 cm^−1^) of untreated and treated CaCO_3_ particles.

The ATR-FTIR and Raman spectra of the CaCO_3_ samples are shown in Figure 4. In Figure 4a, the ATR-FTIR spectra of both CaCO_3_ particle types show the typical bands of vaterite, corresponding to doubly degenerate in-plane OCO deformation bending and CO_3_ out-of-plane bending at 745 and 875 cm^−1^, respectively [36]. In contrast, the characteristic peak of calcite was not observed at 713 cm^−1^, corresponding to the doubly degenerate in-plane OCO bending deformation [43]. The Raman spectra in Figure 4b support the findings of the FTIR spectra. The typical peaks of the vaterite polymorph appear at 744, 1076, and 1089 cm^−1^, corresponding to the ν_4_ in-plane bending, ν_1_ symmetric stretching, and ν_3_ symmetric stretching of CO_3_^2−^, respectively [44,45]. Furthermore, the characteristic peak of calcite at 711 cm^−1^ (ν_4_ in-plane bending) was absent.

**Figure 5:**
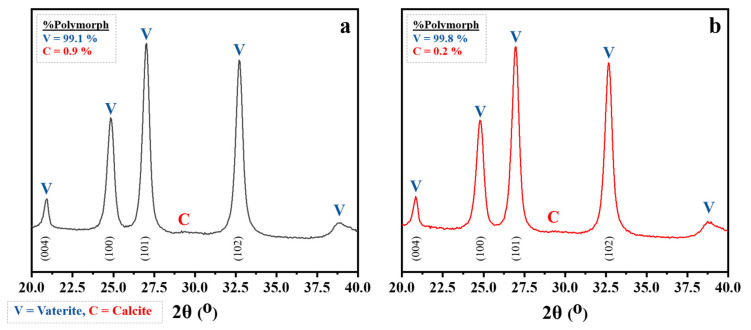
XRD patterns of the obtained CaCO_3_ powders: (**a**) untreated CaCO_3_ and (**b**) treated CaCO_3_, with calculated percentages of the polymorphic phases via Rietveld refinement using MAUD software.

The XRD patterns of untreated and treated CaCO_3_ powders were investigated in the 2θ range of 20–40°, as shown in Figure 5a,b, respectively. The typical peaks of vaterite located at 20.0°, 24.9°, 27.0°, and 32.7° are attributed to the reflections of the (004), (100), (101), and (102) crystalline planes, respectively [46]. Additionally, the typical peak of the calcite polymorph at 29.3° was not found [47]. Powder diffraction analysis coupled with Rietveld analysis (using the MAUD software) is a powerful technique to clarify the structure and polymorphic phase content of powders. The XRD refinement of untreated spherical CaCO_3_ powder revealed a volume fraction of 99.1% for vaterite and 0.9% for calcite, whereas the treated CaCO_3_ powder contained 99.8% vaterite and 0.2% calcite. The minimization of the particle size and maximization of the vaterite content depend strongly on the use of sucrose as an aqueous medium [20,48]. The results confirmed that adding olive soap in the mixing system resulted in a hydrophobic powder and provided high content of the vaterite phase.

**Figure 6:**
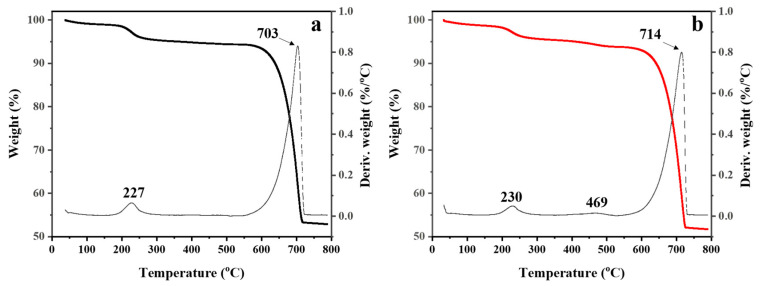
TGA thermograms (black and red solid lines) and DTG curves (dashed line) of (**a**) untreated CaCO_3_ and (**b**) treated CaCO_3_ powders.

The thermal properties of the CaCO_3_ powders were examined by TGA, as presented in Figure 6. The mass change of the sample during heating as a function of temperature (40–800 °C) was demonstrated in TGA thermograms and derivative thermogravimetric (DTG) curves. For both the untreated (Figure 6a) and treated CaCO_3_ (Figure 6b), the decomposition started around 200 °C and was completed at 250 °C, with weight losses of 3–4%, which may have arisen from the decomposition of the sucrose used as an aqueous medium in the preparation process. In the case of hydrophobic CaCO_3_ powder, the decomposition of organic fuel in the soap occurred in the range of 400–500 °C (see Figure 6b). Moreover, the decomposition of CaCO_3_ to CaO and CO_2_ occurred for both the untreated and treated samples in the range of 650–750 °C [49].

### 3.2. NR/Untreated CaCO_3_ and NR/Treated CaCO_3_ Composites

**Figure 7:**
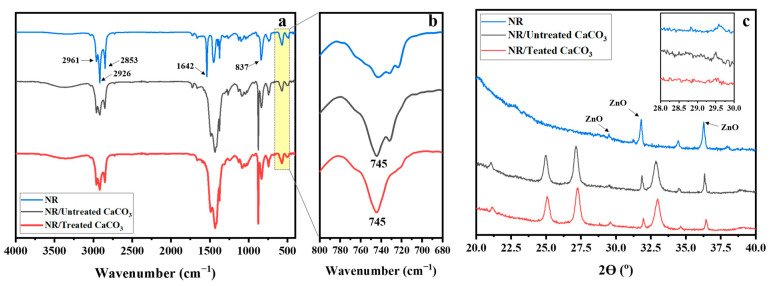
ATR-FTIR spectra of neat NR and NR/CaCO_3_ composites (at filler loading of 20 phr) in the range of wavenumbers (**a**) 400–4000 cm^−1^ and (**b**) 680–800 cm^−1^, and (**c**) X-ray diffractogram of NR and NR/CaCO_3_ composite at filler loading of 20 phr.

The NR/untreated CaCO_3_ and NR/treated CaCO_3_ composites were prepared, and their characteristics were analyzed by ATR-FTIR spectroscopy. Figure 7a shows the ATR-FTIR spectra of neat NR and NR/CaCO_3_ composites filled with 20 phr of CaCO_3_. The chemical structure of NR comprises cis 1,4-polyisoprene. The characteristic peaks of the isoprene unit in NR were found at 837, 1642, 2853, 2926, and 2961 cm^−1^, assigned to the =C-H out-of-plane deformation, C=C stretching, CH_2_ stretching, CH_3_ stretching, and =C-H stretching, respectively. The FTIR spectra of NR/CaCO_3_ composites exhibited an additional peak of CaCO_3_ at 745 cm^−1^ (Figure 7b), indicating the typical band of vaterite for doubly degenerate in-plane OCO. As a result, the vaterite polymorph of CaCO_3_ incorporated in NR latex in the preparation of NR/CaCO_3_ compounds did not convert into the more thermodynamically stable form of calcite. This is because the negative charges of NR particles limit the polymorphic phase transformation of CaCO_3_ fillers [20]. The XRD patterns of CaCO_3_ fillers incorporated in NR are shown in Figure 7c. The typical crystalline phases of vaterite CaCO_3_ were present at 21.0°, 24.9°, 27.1°, and 32.8°. Meanwhile, the typical calcite peak in the range of 28–29° was not detected. Additionally, peaks were observed at 29.5°, 31.5°, and 36.3° for the ZnO that was used in the curing agents. The FTIR and XRD results agree well, demonstrating that no characteristic peaks of calcite were found in the rubber/CaCO_3_ composites.

**Figure 8:**
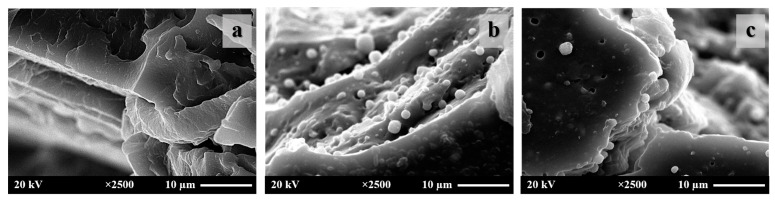
SEM images of the tensile-fractured surface at 2500×, with 10 µm scale bar, of (**a**) neat NR, (**b**) NR/untreated CaCO_3_ composite (20 phr), and (**c**) NR/treated CaCO_3_ composite (20 phr).

The morphologies of the tensile-fractured surfaces of neat NR and NR/CaCO_3_ composites at 20 phr filler loading are shown in Figure 8. Figure 8a shows the smooth surface of the NR matrix without filler. The NR/untreated CaCO_3_ (Figure 8b) and NR/treated CaCO_3_ (Figure 8c) morphological surfaces exhibit both smooth surfaces from the rubber and roughness from the CaCO_3_ particles, and they maintained their initial spherical shape and vaterite polymorph in NR without any transformation. 

**Figure 9:**
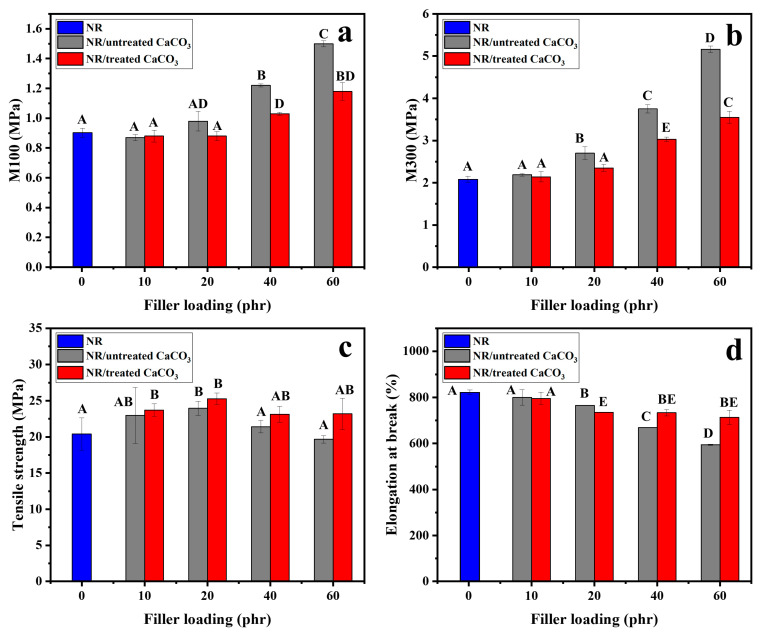
Mechanical properties of neat NR, NR/untreated CaCO_3_, and NR/treated CaCO_3_ composites for filler loadings of 0–60 phr: (**a**) M100, (**b**) M300, (**c**) elongation at break, and (**d**) tensile strength. The results were analyzed by ANOVA, and the different letters (A, AB, C, D, E, AD, BD, BE) above bars refer to significant differences among the means, *p* < 0.05.

The mechanical properties of NR filled with untreated and treated spherical CaCO_3_ powders at different loadings (0, 10, 20, 40, and 60 phr) were investigated, including the 100% and 300% moduli, tensile strength, elongation at break, and hardness. The 100% (M100) and 300% (M300) moduli of neat NR, NR/untreated CaCO_3_, and NR/treated CaCO_3_ composites are presented in Figure 9a,b, respectively. The modulus is an indication of the relative stiffness of the composite. The incorporation of 10 phr CaCO_3_ filler did not result in a significant change in the moduli of the composites. However, increasing the filler loading increased the moduli of the rubber composites, which can be ascribed to the rigidity of the CaCO_3_. Notably, NR/untreated CaCO_3_ exhibited a higher modulus than NR/treated CaCO_3_ composites, which may be attributed to an effect of the soap molecules.

The tensile strengths of NR/CaCO_3_ composites with different filler loadings (0–60 phr) are presented in Figure 9c, as analyzed by ANOVA. The tensile strength of neat NR was 20.35 ± 2.22 MPa. After adding spherical CaCO_3_ powders to the NR, the tensile strength was significantly increased compared with the neat NR (as shown using the different letters above the bars). This result is contrary to the use of CaCO_3_ in plastic, which decreases the tensile strength [50]. Moreover, in the case of the same filler content, the tensile strength of untreated and treated CaCO_3_ added to NR results in significantly different elongation at break. The addition of untreated CaCO_3_ powder at 20 phr provided the highest tensile strength value (23.95 ± 0.97 MPa). Further increases in the filler loading of untreated CaCO_3_ to 40 and 60 phr decreased the tensile strength. This may be due to the poor dispersion of the filler in the rubber composites owing to the polarity difference between NR and CaCO_3_. This can also be explained by the non-homogeneous distributions for high filler concentrations in the rubber matrix, possibly resulting in filler aggregation, which can cause stress concentration and accelerate failure under tension. The tensile strength of the NR/treated CaCO_3_ composites was higher than that of the NR/untreated CaCO_3_ composite at all filler loadings. This may be due to the improved interfacial interaction between the NR and treated CaCO_3_. The highest tensile strength was obtained for NR/treated CaCO_3_ composites with 20 phr loading (25.28 ± 0.81 MPa). Similarly, Y. Yu and coworkers loaded NR with silanized silica-encapsulated CaCO_3_ or untreated CaCO_3_ [51]. Their results showed that the tensile strength of the NR/silanized silica-encapsulated CaCO_3_ vulcanizate was improved compared with the NR/CaCO_3_ vulcanizate, which was attributed to the enhancements in the interfacial compatibility and dispersion of the CaCO_3_ in the rubber matrix. Herein, when the treated CaCO_3_ loading was increased further to 40 and 60 phr, the tensile strength was slightly decreased but still significantly higher than that for the NR/untreated CaCO_3_. The decreased tensile strength at high filler loadings may be due to filler–filler aggregation.

Regarding the elongation of NR/CaCO_3_ composites, as shown in Figure 9d, there was a decrease in the strain at break in NR composites loaded with the CaCO_3_ filler. The elongation at break for neat NR was 821.63 ± 10.25%. For 10 phr of CaCO_3_ particles, the elongation at break of the filled NR composites was similar to that of the neat NR. This is because the low amount of the filler did not disrupt the elongation of rubber chain entanglements during extension. Increasing the filler loading to 20, 40, and 60 phr gradually decreased the elongation at break in the NR/filler composites. The elongation at break decreased to 594.06 ± 3.24% in NR/untreated CaCO_3_ composites and 713.36 ± 31.56% in NR/treated CaCO_3_ composites at 60 phr loading. These results indicate that the addition of rigid inorganic particles reduced the ductility of the rubber composites. In addition, the formation of filler–filler aggregation may have increased the discontinuity of the rubber matrix, resulting in reduced elongation. Furthermore, the elongation of NR/treated CaCO_3_ composites was higher than that of the NR/untreated CaCO_3_ composites at all filler loadings. This can be explained by the presence of the soap on the surface of the treated CaCO_3_, which can serve as a lubricant to facilitate the flow of rubber chains during elongation. Moreover, the spherical-shaped CaCO_3_ treated with olive soap did not show significantly decreased elongation at break even when a large amount (40–60 phr) of the filler was added.

**Figure 10:**
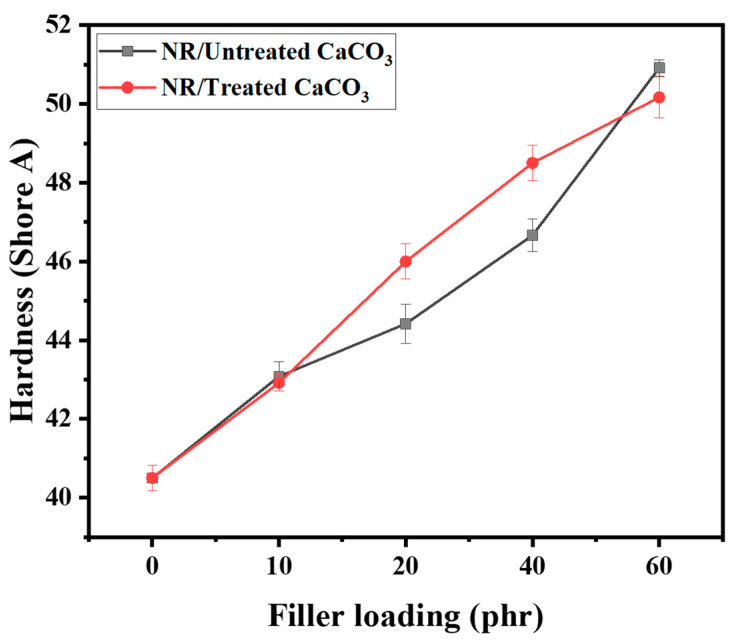
Shore A hardness of NR/CaCO_3_ composites.

Figure 10 shows the Shore A hardness values of neat NR and NR/CaCO_3_ composites. Generally, the Shore A hardness is used to evaluate the resistance of rubbers and elastomers to indentation. Higher values imply greater hardness, while lower values indicate softer materials. Therefore, it is not surprising that the hardness of NR composites increased when the CaCO_3_ filler content increased. The Shore A hardness of neat NR was 40.5 ± 0.3, but the Shore A hardness increased to 50.9 ± 0.2 for NR/untreated CaCO_3_ and 50.2 ± 0.5 for NR/treated CaCO_3_ composites at a loading of 60 phr. At 20 and 40 phr loadings, the Shore A hardness values of NR/treated CaCO_3_ were higher than those of NR/untreated CaCO_3._ This is attributed to the enhanced distribution of the treated CaCO_3_ filler in the rubber matrix compared with the case of untreated CaCO_3_.

**Figure 11:**
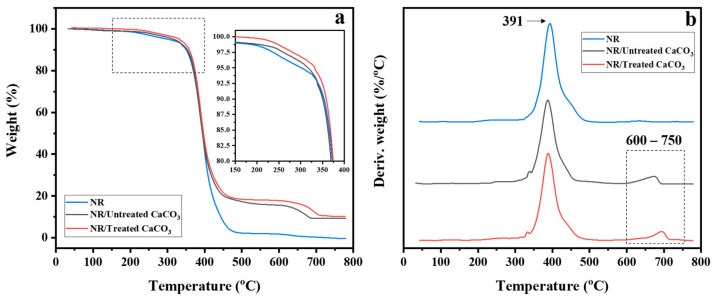
(**a**) TGA curves and (**b**) DTG curves of neat NR, NR/untreated CaCO_3_, and NR/treated CaCO_3_ composites at 20 phr loading in the temperature range of 40–800 °C, with scanning rate of 20 °C min^−1^.

The thermal decomposition profiles of neat NR, NR/untreated CaCO_3_, and NR/treated CaCO_3_ samples at 20 phr filler loading in terms of TGA curves (Figure 11a) and DTG curves (Figure 11b) were determined. The NR revealed significant degradation in the range of 360–450 °C, which was ascribed to the polymer’s pyrolysis, with a peak at 391 °C. NR filled with CaCO_3_ powders exhibited major degradation in a similar range, resulting in weight loss of approximately 65% due to the decomposition of NR. A second stage (650–750 °C) of thermal decomposition appeared for NR/CaCO_3_ composites, with weight losses of 16–20%. In addition, in the range of 200–320 °C, the weight loss of the NR was slightly higher than that of the NR/CaCO_3_ composites. The results suggest that CaCO_3_ improved the thermal resistance of the rubber composites. This result is consistent with the work of Umunakwe et al., who investigated NR filled with carbon black and calcium carbonate. They demonstrated that calcium carbonate could improve the thermal resistance of the rubber vulcanizate compared with the carbon black-filled rubber [52].

## 4. Conclusions

Spheroidal-shaped CaCO_3_ particles with and without surface treatment were easily synthesized by precipitation using sucrose as an aqueous medium to reduce the particle size to the submicron scale, and olive soap was used to modify the surface properties of the CaCO_3_ particles. The optical contact angle of CaCO_3_ without treatment was 16.5°, which reflects the general nature of CaCO_3_, while that of treated CaCO_3_ was 145.4°, which is considered hydrophobic. The CaCO_3_ particles were larger and rougher after treatment with olive soap because of the soap molecules adsorbed on the surfaces of CaCO_3_ particles. The percentage of the vaterite crystalline phase was higher than 99% for both the untreated and treated CaCO_3_ powders. The mechanical properties of NR vulcanizates were enhanced when CaCO_3_ fillers (0–60 phr) were applied. The NR/treated CaCO_3_ composites provided higher tensile strength than the NR/untreated CaCO_3_ composites for all variations of filler loading. This may have been due to the improved interfacial interaction between the NR and hydrophobic CaCO_3_. The filler loading of 20 phr was optimal in obtaining the highest tensile strength in the rubber composites. In addition, the elongation ability of the NR/treated CaCO_3_ only decreased slightly, while the hardness of the NR composites was significantly improved_._ In summary, spherical CaCO_3_ particles can be used to effectively reinforce and improve the mechanical properties of NR. The facile method for the surface modification of the spherical-shaped CaCO_3_ using olive soap resulted in enhanced hardness and tensile strength. These findings can broaden the application of CaCO_3_ in polymer composites.

## Figures and Tables

**Table 1:** Mixing formulation of NR/CaCO_3_ composites.

Chemicals	Content (phr *)
NR	100
CaCO_3_	0, 10, 20, 40, 60
ZnO	1.8
Stearic acid	2.0
CBS	1.0
Sulfur	1.5

* phr, parts per hundred rubber.

**Table 2:** Particle size, BET-specific surface area, contact angle, and polymorphic phase percentage of obtained CaCO_3_.

Types of CaCO_3_	Particle Size (µm) *	BET Value (m^2^/g)	Contact Angle (°)	Polymorphic Phase (%)	
				Vaterite	Calcite
Untreated	0.42 ± 0.14	39.32	16.5	99.1	0.9
Treated	0.52 ± 0.16	34.45	145.4	99.8	0.2

* The data are presented as means ± standard deviations.

## Data Availability

The data presented in this research are available on request from the corresponding author.

## References

[1] Phinyocheep P., Phetphaisit C.W., Derouet D., Campistron I., Brosse J.C. (2005). Chemical degradation of epoxidized natural rubber using periodic acid: Preparation of epoxidized liquid natural rubber. J. Appl. Polym. Sci..

[2] Phinyocheep P., Kohjiya S., Ikeda Y. (2014). 3—Chemical modification of natural rubber (NR) for improved performance. Chemistry, Manufacture and Applications of Natural Rubber.

[3] Singh M. (2018). The Colloidal Properties of Commercial Natural Rubber Latex Concentrates. J. Rubber Res..

[4] Fisher H.L. (1939). Vulcanization of rubber vulcanization of rubber. Ind. Eng. Chem..

[5] Leblanc J.L. (2002). Rubber–filler interactions and rheological properties in filled compounds. Prog. Polym. Sci..

[6] Rothon R., Rothon R. (2017). Particulate Fillers in Elastomers. Fillers for Polymer Applications.

[7] Turku I., Kärki T. (2013). Research progress in wood-plastic nanocomposites: A review. J. Thermoplast. Compos. Mater..

[8] Kim J.H., Jeong H.Y. (2005). A study on the material properties and fatigue life of natural rubber with different carbon blacks. Int. J. Fatigue.

[9] Kaliyathan A.V., Rane A.V., Huskic M., Kunaver M., Kalarikkal N., Rouxel D., Thomas S. (2020). Carbon black distribution in natural rubber/butadiene rubber blend composites: Distribution driven by morphology. Compos. Sci. Technol..

[10] Robertson C.G., Hardman N.J. (2021). Nature of Carbon Black Reinforcement of Rubber: Perspective on the Original Polymer Nanocomposite. Polymers.

[11] Sarkawi S.S., Dierkes W.K., Noordermeer J.W.M. (2014). Reinforcement of natural rubber by precipitated silica: The influence of processing temperature. Rubber Chem. Technol..

[12] Fang Q., Song B., Tee T.-T., Sin L.T., Hui D., Bee S.-T. (2014). Investigation of dynamic characteristics of nano-size calcium carbonate added in natural rubber vulcanizate. Compos. Part. B Eng..

[13] Lay M., Hamran N., Rashid A.A. (2019). Ultrafine calcium carbonate-filled natural rubber latex film: Mechanical and post-processing properties. Iran. Polym. J..

[14] Sadeghi Ghari H., Jalali-Arani A. (2016). Nanocomposites based on natural rubber, organoclay and nano-calcium carbonate: Study on the structure, cure behavior, static and dynamic-mechanical properties. Appl. Clay Sci..

[15] Akbari A., Jawaid M., Hassan A., Balakrishnan H. (2013). Epoxidized natural rubber toughened polylactic acid/talc composites: Mechanical, thermal, and morphological properties. J. Compos. Mater..

[16] Khan I., Bhat A.H., Thomas S., Maria H.J., Joy J., Chan C.H., Pothen L.A. (2013). Micro and Nano Calcium Carbonate Filled Natural Rubber Composites and Nanocomposites. Natural Rubber Materials, Volume 2: Composites and Nanocomposites.

[17] Konopacka-łyskawa D., Lackowski M. (2011). Influence of ethylene glycol on CaCO_3_ particles formation via carbonation in the gas–slurry system. J. Cryst. Growth.

[18] Pérez-Villarejo L., Takabait F., Mahtout L., Carrasco-Hurtado B., Eliche-Quesada D., Sánchez-Soto P.J. (2018). Synthesis of vaterite CaCO_3_ as submicron and nanosized particles using inorganic precursors and sucrose in aqueous medium. Ceram. Int..

[19] Gibaud A., Younas D., Matthews L., Narayanan T., Longkaew K., Hageberg I.U., Chushkin Y., Breiby D.W., Chattopadhyay B. (2023). Insights into the precipitation kinetics of CaCO_3_ particles in the presence of polystyrene sulfonate using in situ small-angle X-ray scattering. J. Appl. Crystallogr..

[20] Longkaew K., Tessanan W., Daniel P., Phinyocheep P., Gibaud A. (2023). Using sucrose to prepare submicrometric CaCO_3_ vaterite particles stable in natural rubber. Adv. Powder Technol..

[21] Baqiya M.A., Lailiyah Q., Riyanto A., Arifin Z., Triwikantoro, Zainuri M., Pratapa S., Darminto (2020). Precipitation Process of CaCO_3_ from Natural Limestone for Functional Materials. J. AOAC Int..

[22] Owuamanam S., Cree D. (2020). Progress of Bio-Calcium Carbonate Waste Eggshell and Seashell Fillers in Polymer Composites: A Review. J. Compos. Sci..

[23] Rodriguez-Blanco J.D., Shaw S., Benning L.G. (2011). The kinetics and mechanisms of amorphous calcium carbonate (ACC) crystallization to calcite, viavaterite. Nanoscale.

[24] Boyjoo Y., Pareek V.K., Liu J. (2014). Synthesis of micro and nano-sized calcium carbonate particles and their applications. J. Mater. Chem. A.

[25] Jimoh O.A., Ariffin K.S., Hussin H.B., Temitope A.E. (2018). Synthesis of precipitated calcium carbonate: A review. Carbonates Evaporites.

[26] Nehrke G., Van Cappellen P. (2006). Framboidal vaterite aggregates and their transformation into calcite: A morphological study. J. Cryst. Growth.

[27] Bragg W.L. (1997). The structure of aragonite. Proc. R. Soc. London Ser. A Contain. Pap. A Math. Phys. Character.

[28] Kogo M., Suzuki K., Umegaki T., Kojima Y. (2021). Control of aragonite formation and its crystal shape in CaCl_2_-Na_2_CO_3_-H_2_O reaction system. J. Cryst. Growth.

[29] Tas A.C. (2009). Monodisperse Calcium Carbonate Microtablets Forming at 70 °C in Prerefrigerated CaCl2–Gelatin–Urea Solutions. Int. J. Appl. Ceram. Technol..

[30] Lu J., Ruan S., Liu Y., Wang T., Zeng Q., Yan D. (2022). Morphological characteristics of calcium carbonate crystallization in CO_2_ pre-cured aerated concrete. RSC Adv..

[31] Utrera-Barrios S., Perera R., León N., Santana M.H., Martínez N. (2021). Reinforcement of natural rubber using a novel combination of conventional and in situ generated fillers. Compos. Part C Open Access.

[32] Zhao R., Yin Z., Zou W., Yang H., Yan J., Zheng W., Li H. (2023). Preparation of High-Strength and Excellent Compatibility Fluorine/Silicone Rubber Composites under the Synergistic Effect of Fillers. ACS Omega.

[33] Surya I., Sinaga R. (2019). Tensile and rheometric properties of calcium carbonate-filled natural rubber compounds without/with lauryl alcohol. IOP Conf. Ser. Mater. Sci. Eng..

[34] Shi X., Rosa R., Lazzeri A. (2010). On the Coating of Precipitated Calcium Carbonate with Stearic Acid in Aqueous Medium. Langmuir.

[35] Cao Z., Daly M., Clémence L., Geever L.M., Major I., Higginbotham C.L., Devine D.M. (2016). Chemical surface modification of calcium carbonate particles with stearic acid using different treating methods. Appl. Surf. Sci..

[36] Abidi L., Amiard F., Delorme N., Ouhenia S., Gibaud A. (2022). Using saponified olive oil to make cost effective calcium carbonate particles superhydrophobic. Adv. Powder Technol..

[37] Subagjo, Wulandari W., Adinata P.M., Fajrin A. (2017). Thermal decomposition of dolomite under CO_2_-air atmosphere. AIP Conf. Proc..

[38] Erickson K.L. Application of Low-Heating Rate TGA Results to Hazard Analyses Involving High-Heating Rates. Proceedings of the SAMPE’08 Conference.

[39] Koeipudsa N., Chanthateyanonth R., Daniel P., Phinyocheep P. (2022). Development of natural rubber nanocomposites reinforced with cellulose nanocrystal isolated from oil palm biomass. J. Polym. Res..

[40] (2002). Standard Test Methods for Vulcanized Rubber and Thermoplastic Rubbers and Thermoplastic Elastomers-Tension.

[41] (2015). Standard Test Method for Rubber Property—Durometer Hardness.

[42] Rodrigues N., Casal S., Pinho T., Cruz R., Peres A.M., Baptista P., Pereira J.A. (2021). Fatty Acid Composition from Olive Oils of Portuguese Centenarian Trees Is Highly Dependent on Olive Cultivar and Crop Year. Foods.

[43] Reig F.B., Adelantado J.V., Moya Moreno M.C. (2002). FTIR quantitative analysis of calcium carbonate (calcite) and silica (quartz) mixtures using the constant ratio method. Application to geological samples. Talanta.

[44] Dupont L., Portemer F., late Michel Figlarz T. (1997). Synthesis and study of a well crystallized CaCO_3_ vaterite showing a new habitus. J. Mater. Chem..

[45] Trushina D.B., Bukreeva T.V., Kovalchuk M.V., Antipina M.N. (2014). CaCO_3_ vaterite microparticles for biomedical and personal care applications. Mater. Sci. Eng. C Mater. Biol. Appl..

[46] Yang L.-F., Chu D.-Q., Sun H.-L., Ge G. (2016). Room temperature synthesis of flower-like CaCO_3_ architectures. New J. Chem..

[47] Thriveni T., Nam S.Y., Ahn J.-W., Um N. Enhancement of arsenic removal efficiency from mining waste water by accelerated carbonation. Proceedings of the IMPC 2014—27th International Mineral Processing Congress.

[48] Konopacka-Łyskawa D., Czaplicka N., Kościelska B., Łapiński M., Gębicki J. (2019). Influence of Selected Saccharides on the Precipitation of Calcium-Vaterite Mixtures by the CO_2_ Bubbling Method. Crystals.

[49] Abdolmohammadi S., Siyamak S., Ibrahim N.A., Yunus W.M.Z.W., Rahman M.Z.A., Azizi S., Fatehi A. (2012). Enhancement of Mechanical and Thermal Properties of Polycaprolactone/Chitosan Blend by Calcium Carbonate Nanoparticles. Int. J. Mol. Sci..

[50] Buasri A., Chaiyut N., Borvornchettanuwat K., Chantanachai N., Thonglor K. (2012). Thermal and Mechanical Properties of Modified CaCO_3_/PP Nanocomposites. World Acad. Sci. Eng. Technol. Int. J. Chem. Mol. Nucl. Mater. Metall. Eng..

[51] Yu Y., Zhang J., Wang H., Xin Z. (2020). Silanized Silica-Encapsulated Calcium Carbonate@Natural Rubber Composites Prepared by One-Pot Reaction. Polymers.

[52] Umunakwe R., Oyetunji A., Adewuyi B., Eze W., Nwigwe S., Umunakwe I. (2019). Mechanical properties and microstructure of hybrid vulcanized natural rubber filled with carbon black and Nano-CaCO_3_ from achatina achatina shells. J. Met. Mater. Miner..

